# Common sense, scientific images, and the aesthetic mode of knowing

**DOI:** 10.1007/s40656-025-00697-z

**Published:** 2025-10-09

**Authors:** Shani Inbar, Eva Jablonka, Simona Ginsburg, Anna Zeligowski

**Affiliations:** 1 Department of Natural Science, The Open University of Israel, POB 808, 4353701 Raanana, Israel; 2 Cohn Institute for the History and Philosophy of Science and Ideas, Tel Aviv University, 69978 Tel Aviv, Israel; 3 Bari, Italy

**Keywords:** Common sense, Scientific representations, Kantian aesthetics, Normative commitments

## Abstract

In modern English, common sense refers to an intuitive capacity to grasp self-evident truths and make judgments that require no special training or expertise. Although often treated as universal and ahistorical, its standing as an epistemic authority, especially within the sciences, has been contested, revised, and reconfigured over the past two centuries. Yet scientists’ assumptions about the reliability of common sense typically remain implicit, embedded in a normative background that is rarely examined but quietly guides scientific thought. This paper examines how different attitudes toward common sense are reflected in the aesthetic choices and visual references scientists use. Through three case studies—Ernst Haeckel, Conrad Waddington, and Ginsburg & Jablonka—we demonstrate how their respective views, firmly rooted in their historical context, are made accessible through their aesthetic choices. Examining these choices reveals that scientific images, particularly those with artistic qualities, do more than depict scientific knowledge; they reflect underlying normative commitments, shaping what is seen as intelligible and scientifically meaningful. They are sites where scientific sensibilities and epistemic commitments become visible and available for critique. Drawing on Kant’s notion of sensus communis, we suggest that aesthetic judgments, particularly of scientific representations, provide a reflective standpoint from which such implicit commitments can be evaluated.

## Introduction

In *Common Sense: A Political History* (2011), Sophia Rosenfeld writes:

In modern parlance, we sometimes use common sense to mean the basic human faculty that lets us make elemental judgments about everyday matters based on everyday, real-world experience […] Other times we mean the widely shared and seemingly self-evident conclusions drawn from this faculty, the truisms about which all sensible people agree without argument or even discussion. (p. 1)

For much of the eighteenth and nineteenth centuries, many branches of science operated within this framework, believing common sense offered a reliable guide to reality. They trusted this faculty, and the ontologies they proposed remained accessible to ordinary reasoning. Nature was assumed to be structured in a way that lent itself to order and lawfulness, where, at least, time, space, and causality provided stable scaffolding not only for everyday perception but also for scientific theorizing. However, in certain domains, such as mathematics, thermodynamics, and electromagnetism, scientific developments increasingly diverged from common sense intuitions, signaling early tensions between conceptual innovation and intuitive accessibility.

By the early twentieth century, confidence in common sense had eroded even further as scientific theories increasingly departed from intuitive understanding. The rise of quantum mechanics, in particular, dismantled the very foundations of common sense. Time and space were no longer seen as separate, uniformly measurable quantities, but as parts of a unified four-dimensional continuum. Even more unsettling was the realization that reality itself was not independent of observation and, suddenly, the basic constituents of the world were not static “bits of matter” but dynamic processes described in probabilistic terms. Cats and stones were no longer stable objects but fluctuating processes capable of vanishing and reappearing elsewhere or existing simultaneously in multiple locations. Intuitive notions about time, space, and causality seemed to break down, and science gradually distanced itself from common sense, treating it as unreliable and misleading.

The devastation of World War I brought this tension to a breaking point, not only as a geopolitical catastrophe, but as a crisis of moral and epistemological disillusionment. It revealed a world governed not by reason but by prejudice and irrationality, signaling the collapse of shared meaning and consensual truths. This loss of faith contributed to a desire to replace the world, escape the human condition, and reject commonsensical intuitions that seemed to offer nothing but disaster and suffering.

But the rejection of common sense came at a cost. Hannah Arendt (1958) referred to it as “world alienation”—the severing of knowledge from perceptual experience that fractured the shared, familiar world and, with it, science’s role in public life. The crisis deepened in the wake of World War II, which not only intensified skepticism about human rationality but also ushered in new disciplinary shifts. Developments in anthropology and social psychology challenged static, universal conceptions of human nature, emphasizing context-dependent modes of reasoning. At the same time, philosophical critiques of science, such as Ludwik Fleck’s analysis of how scientific facts are constructed and, later, Thomas Kuhn’s influential theory of paradigm shifts, further undermined faith in the trans-historical stability of the ‘common understanding.’

This ‘loss of a shared world’ has had political consequences that extend beyond what Arendt anticipated. In this climate, certain political movements appeal to common sense as the only remaining source of clarity—as a basis for concrete solutions that intellectuals, experts, and scientists seem unable to provide. Scientific discourse, with its abstract language and theoretical models, appears remote, obscure, and disconnected from real-world concerns, and there is a renewed call for common sense as a reaction against abstraction and elitism.

As a result, science becomes increasingly vulnerable to denial and rejection—both internally, because scientists themselves recognize the normative and constructed nature of their frameworks, and externally, because they face a public that demands clarity and intuitive coherence. In this environment, common sense walks a fine line: it can foster shared understanding, but it can also limit the space of innovation. Science operates within this tension, continually negotiating its relationship to intuitive reasoning and public trust.

This fluctuation—from trust in the nineteenth century to rejection in the 20th, to ambivalence in the 21st—is not just a shift in opinion; it reflects deeper normative commitments that run beneath explicit scientific debate. The status of common sense shapes which ontologies science can accommodate, what counts as obvious, and what kinds of explanations seem intelligible. These commitments are seldom made explicit, yet they often surface in scientific imagery. Scientific representations, in this sense, are not neutral explanatory tools; they encode assumptions, values, and explanatory preferences. They shape how explanations are framed, they privilege specific perspectives, and they naturalize some ways of seeing.

Let’s consider three images: The first is Ernst Haeckel’s (1904) lithograph of *Discomedusae* in his *Kunstformen der Natur* (plate 8), which depicts a meticulously detailed, symmetrical arrangement of jellyfish belonging to this order. At its center, the large and ornate *Annasethe* (named after his first wife) dominates the composition. Beneath its umbrella-like pattern with its delicate scalloped edges, clusters of frilled oral arms and slender tentacles twist and curl. Flanking *Annasethe* are two other species—*Chrysaora mediterranea* at the lower right and *Floscula promethea* at the upper left—each depicted with similarly intricate textures: ribbed bells, streaming tentacles, and elaborately branched oral arms. In reality, these medusae differ significantly in size, but Haeckel adjusted their proportions to create a balanced and harmonious design of these marine organisms.

The second image is Yolanda Sonnabend’s *Epigenetic Landscape* in C.H. Waddington’s *Tools for Thought* (1977), Fig. 7b.7. It depicts an abstract, almost calligraphic rendering of a branching terrain, drawn in sweeping, fluid brush-like strokes that fold into ridges and valleys. The sloping surface resembles a hillside and several small spheres are scattered across the scene, some nestled within the channels of the landscape, others seemingly suspended above it. Their placement suggests movement from higher to lower positions, or from a state of suspension to one of settlement. Set against an empty background, the dark, shifting surface gives the scene a distinctly symbolic quality, setting it apart from the ordinary world.

**Fig. 1:**
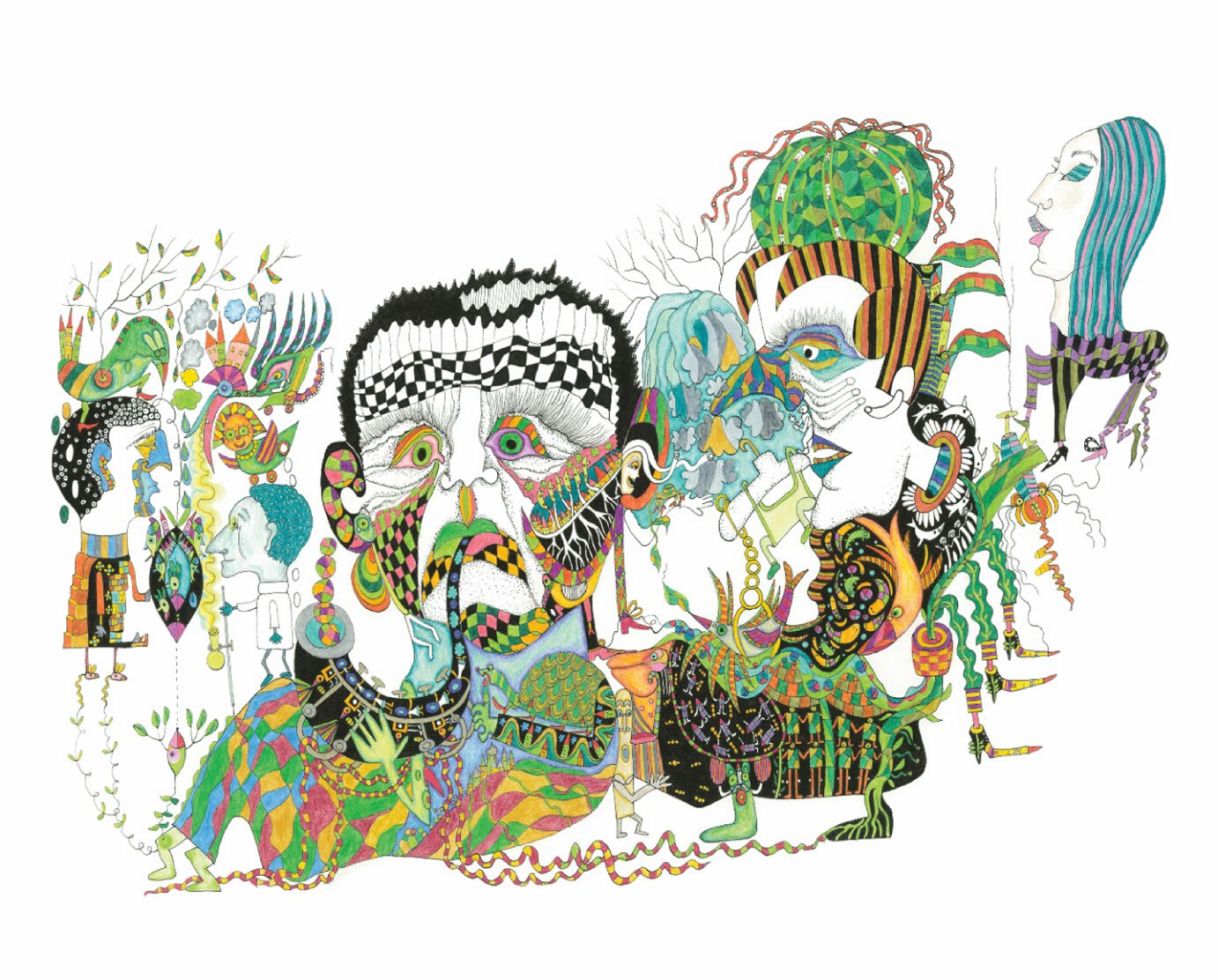
Anna Zeligowski*, On Some Peculiarities of the Symbolic Species,* in Ginsburg and Jablonka’s *Picturing the Mind through the Lens of Evolution* (2022). Permission given by the artist (@ Anna Zeligowski)

The last image is titled *On Some Peculiarities of the Symbolic Species* from Ginsburg and Jablonka’s *Picturing the Mind through the Lens of Evolution* (2022). It shows a lively, colorful scene filled with human- and animal-like figures. Each figure wears a different facial expression—wonder, apathy, dread, or self-importance—suggesting an inner world visible only through these gestures. Though they seem loosely connected within the same space, each figure appears as if absorbed in its own separate world. The drawing is rich with small details that invite closer inspection, and the overall effect is both playful and slightly enigmatic (Fig. 1).

These images constitute the synopsis of our story and show the broader shifts in the role of imagery in science, particularly in the biological sciences and evolutionary developmental studies. Each reflects a distinct aesthetic code, points to different epistemic values of images in science, and encodes a particular type of scientific perspective. This sequence has a historical rhythm: it is no accident that Haeckel’s *Discomedusae* appear as they do—a naturalistic, almost intuitive representation; that Sonneband’s image is cryptic and abstract; and that Zeligowski’s ‘peculiarities’ strikes an eccentric yet captivating balance. Each image evokes different epistemological sensibilities and reflects the evolving relationship between science and visual representation. They reveal distinct senses of understanding—whether grounded in common sense, scientific abstraction, or a more creative engagement with complexity.

The contrast between these representations illustrates how the authority of common sense in science has been repeatedly reconfigured. Haeckel’s work exemplifies a time when scientific inquiry remained closely aligned with common understanding. His meticulous, highly symmetrical drawings suggest a world in which nature’s order is immediately graspable, structured in ways that resonate with everyday perception. Through such images, knowledge remained anchored in the familiar, reinforcing a sense of continuity between intuitive reasoning and scientific discovery.

By contrast, Waddington’s use of artistic illustrations marks a decisive break from this tradition. Here, scientific knowledge is grounded not in perceptual familiarity, but in abstraction—a world that no longer pays allegiance to common sense. The landscape presents a conceptual model rather than a direct visualization of biological processes, demanding interpretation rather than offering intuitive clarity. Such abstract scientific representations seem to withhold immediate comprehension, separating those initiated into the scientific discourse and those excluded from it. In Arendt’s terms, this is scientific alienation fully realized—where knowledge is no longer part of a shared human world.

Zeligowski’s work introduces a mode of negotiation. Though still abstract, it incorporates figurative elements that render its subject matter more engaging and relatable. It attempts to recover a form of intelligibility that does not rely on the common sense of an earlier scientific era but still seeks to engage the viewer’s perceptual and imaginative faculties. It recognizes that scientific representations are not just explanatory tools but also invitations, bridging the conceptual rigor of science and the intuitive understanding of those who engage with it.

If the history of common sense in science is a history of shifting epistemic commitments, scientific representations offer a unique vantage point into these shifts. They encode historically contingent and normatively loaded judgments about knowledge, reality, and the communicability of scientific ideas. At the same time, these representations reveal that science has always appealed—though in different ways—to something that is, nevertheless, transhistorical—the human ability to make aesthetic judgments. While science has not always trusted common sense as is popularly understood, it has, time and again, appealed to what Kant referred to as the *sensus communis*—a capacity to find non-discursive coherence and communicability in what otherwise remains beyond conceptual reach.

What makes Kant’s account of aesthetic judgment particularly interesting here is its claim to a kind of universality despite being rooted in subjective experience. Aesthetic judgments, on his account, have normatively authoritative force: Their *universal validity* rests on the assumption of a shared capacity for judgment that allows subjective responses to carry the force of claims others ought to accept, even though they are non-conceptual. This is what gives scientific images their normative potential. The very possibility of accessing underlying normative commitments through aesthetic forms depends on this notion. It explains how a particular image could stand for, or gesture toward, a universal that resists conceptual articulation.

This dimension makes scientific representations—especially those with artistic qualities—valuable not only for what they depict but for how they frame and mediate scientific understanding. As our case studies show, they can both engage with and transform normative commitments that guide scientific reasoning. This transformation is possible because aesthetic judgments make normative demands and because they confront us with an alternative, universally valid way of seeing the world. By invoking this aesthetic sensibility, scientific representations do more than illustrate—they invite a kind of orientation toward shared meaning.

In the following, we first outline the major historical shifts in scientists’ commitment to ‘the common human understanding,’^1^ examining how these concepts function as part of the normative backdrop characteristic of their time (Sect. 2). We then turn to our three case studies—Ernst Haeckel (1834–1919), Conrad Waddington (1905–1975), and Ginsburg & Jablonka—and demonstrate how the reputation of common sense is reflected in their use of artistic illustrations (Sect. 3, 4 and 5). We show that each of them used art to both reflect and respond to what they perceived as its role in science. For Haeckel, art was a means of refining common sense and stripping it of dogma and superstition; for Waddington, it was a way to move beyond common sense, constructing new conceptual models for capturing biological complexity; and for Ginsburg & Jablonka, it became a way to render implicit assumptions visible and available for critique, inviting readers to engage with the work and serving as a means of mediating between scientific rigor and shared meaning. These sections are, therefore, titled *Art as an Antidote to Superstition*, *Art as a Substitute for Common Sense*, and *Art as a Mode of Negotiation*, respectively. Finally, we explore Kant’s notion of the *sensus communis* as our motivation for using aesthetic references as guides for uncovering embedded assumptions and as the basis for our analysis of the role of aesthetic judgment in science (Sect. 6). We conclude by positioning our approach within the context of recent attempts to explain the role of aesthetic judgments in science.

While this study focuses on shifting views of ‘the common understanding’ as a key factor in the evolution of scientific representations, other explanations for the apparent differences between these representations hold equal significance. One such explanation lies in how biologists, in each period, have understood and addressed teleology and goal-directedness in biology. From the mechanistic models of the nineteenth century to systems-thinking in the twentieth and twenty-first centuries, scientists have had different views on purpose and design in nature and on concepts such as function and adaptation. Another important factor may be the evolving notions of objectivity and subjectivity in science, particularly regarding the extent to which scientific representations depend on the observer’s perspective and knowledge. However, these explanations are not mutually exclusive. As the following sections will show, they are deeply intertwined, forming a web of commitments that manifest in the aesthetic choices scientists make.

## The reputation of ‘common sense’ as an epistemic authority

Historically, the notion of ‘the good sense of the people’ emerged in the Enlightenment as a response to a crisis of authority that began with the Reformation and continued with the rise of modern science. It was conceived as a new form of authority—a natural, instinctive capacity shared by all people, allowing them to discern fundamental truths without the need for specialized expertise. This idea resonated with democratic ideals, particularly as voting rights expanded throughout the nineteenth century, granting previously marginalized groups—such as working-class men, women, and racial minorities—access to the political sphere. Common sense was invoked to legitimize democracy, suggesting that “the people,” when not misled by false authorities, possessed an infallible, instinctive sense of what is right and true (Rosenfeld, 2011).

However, this period also saw a growing anxiety about the limitations of common sense. It could be invoked either to support efforts toward democratization or to undermine them, to affirm the shared humanity of all people, or to reinforce distinctions based on race, gender, religion, or nationality. As Rosenfeld notes, “The great issue of the nineteenth century has sometimes been said to be democracy and its dangers” (Rosenfeld, 2011, p. 228), particularly concerning the ability of marginalized groups to engage in political judgment. Science played a significant role in these debates, often promoting Xenophobic and racialized distinctions by portraying foreigners, minorities, and colonial subjects as outside the realm of “common” understanding. At the same time, nationalism and imperialism capitalized on the idea of ‘shared understandings’ to foster unity within nations while excluding those deemed outsiders.

World War I marked a turning point—not just as a geopolitical calamity but as a profound crisis of faith in moral and epistemological certainties, exposing the vulnerability of rational discourse and the failure of common sense to sustain shared understandings. In this atmosphere of disenchantment, the idea of ‘the good sense of the people’ appeared irretrievably lost. This disillusionment was not only epistemic but also aesthetic. Nowhere was this more powerfully embodied than in the Dada movement, which emerged as a radical rejection of the cultural and intellectual structures that had led to the war. The Dadaists responded to this crisis by embracing nonsense, irrationality, and absurdity as artistic strategies, exposing the hollowness of inherited norms and the breakdown of any stable, universal meaning (Rosenfeld, 2011). In doing so, they rejected the idea of common understanding as a foundation for knowledge and laid bare the aesthetic dimension of this epistemic rupture.

At the same time, the advent of quantum mechanics profoundly unsettled commonsensical intuitions about the nature of reality. Concepts like space, time, identity, and causality lost their familiar meaning and were radically reevaluated. Time and space were no longer treated as distinct, measurable dimensions, and it became evident that measurement itself could influence the state of a system, challenging the very notion of an objective, observer-independent world. Nature’s basic constituents were now conceived as dynamic processes rather than fixed, immutable objects.

A. N. Whitehead captured this shift in his reflections on the trajectory of scientific thought, observing how natural science gradually dismantled the foundation of common sense notions.

the development of natural science has gradually discarded every single feature of the original common-sense notion. Nothing whatever remains of it, considered as expressing the primary features in terms of which the universe is to be interpreted. The obvious common-sense notion has been entirely destroyed, so far as concerns its function as the basis for all interpretation. One by one, every item has been dethroned. (Whitehead, 1934, 14–15)^2^

This unraveling continued in the human sciences. Anthropology and social psychology introduced a new, context-dependent understanding of human nature. Studies of cultural variation demonstrated that moral values, perceptual categories, and basic cognitive structures were shaped by historical and social conditions rather than by an innate, universal reason. The relativization of human thought reached its height in the 1920s with philosophy’s linguistic turn, which emphasized how language shapes experience, thereby further undermining the notion of a context-independent “common” sense. Ludwik Fleck’s (1935) analysis of the construction of scientific facts, and later Thomas Kuhn’s influential theory of scientific change (Kuhn, 1962), emerged as transformative forces and demonstrated that scientific knowledge was not the gradual accumulation of objective truths but the product of shifting conceptual frameworks. The very notion of human rationality was under scrutiny, revealing that judgments about the world could not be made outside the bounds of language and historically contingent assumptions.

Arendt described this as a rupture of alienation between humans and the world they inhabited. She saw modern science and the scientific image it presented as an attempt to escape the human condition and a desire to replace it with something man has made himself (Arendt, 1998, p. 2). Humans, she wrote, learned that their senses were not suited to comprehend nature, that everyday experience often leads to error and delusion (Arendt, 1961, p. 55), and that the properties of natural phenomena depend on how they are observed. Reality was no longer accessible through direct experience, and the certainty of appearances was replaced with abstract mathematical descriptions. The very structures that once sustained a shared common world had disintegrated. With common sense no longer serving as a stable foundation, both knowledge and political life became unmoored, leaving individuals increasingly detached from a shared, intelligible world (Hirsch, 2020).

In *The Human Condition* (1958), Arendt notes that “What makes mass society so difficult to bear is not the number of people involved…but the fact that the world between them has lost its power to gather them together, to relate them and to separate them” (Arendt, 1998, pp. 52–53). Traditional science played an essential role in building a lasting human artifice of truths that existed independently of human intervention— a stable dwelling place for human life (Undurraga, 2019). These truths, like the durable objects produced through work, were regarded as fixed and enduring: “To live together in the world means essentially that a world of things is between those who have it in common, as a table is located between those who sit around it; the world, like every in-between, relates and separates men at the same time.” (Arendt, 1998, p. 52).

In modern science, Arendt argues, knowing has become inseparable from making (e.g., Hirsch, 2020; Undurraga, 2019). Instead of discovering pre-existing truths, science now produces them, with thought increasingly dominated by the kind of fabrication associated with manufacturing (Hirsch, 2020). On the new notion of Process, Arendt observed that: “actual objects of knowledge can no longer be things of eternal motions but must be processes” (Arendt, 1998, p. 296). This process, she explains, was originally the fabrication process, which ‘disappeared in the product’ (Arendt, 1998, p. 297). However, with modern science’s emphasis on process over product, the focus has shifted away from the end result—the actual ‘table’ around which people sit. “Suddenly, through some magic trick, [people] see the table vanish from their midst” (Arendt, 1998, p. 53). In doing so, science alienated itself from the ‘thing-character of the world’ (Undurraga, 2019), making it ever more difficult for the world to serve as a stable stage for political action.

In today’s world, public life is saturated with discordant voices reacting to fragmented, often contradictory information, making it increasingly difficult to sustain a coherent, collective understanding of reality. In the West, the appeal to ‘common reason’ as the basis for effective political solutions has increasingly been co-opted by some segments of the political right (Rosenfeld, 2011). These appeals offer answers that intellectuals, experts, and scientists cannot provide because their vision is so clouded and their language so obscure. This has made science more susceptible to denial and disbelief (Crease, 2017), finding itself at odds with a public that demands clarity and self-evident truths, and caught between the need for credibility and the risk of alienation.

In the following sections, we turn to three examples—Ernst Haeckel (1834–1919), Conrad Waddington (1905–1975), and Ginsburg & Jablonka—whose work reflects the evolving tensions between common sense, scientific explanation, and aesthetic representation. Their approaches reveal distinct assumptions about the role of visualization in scientific inquiry, serving as a window into the unspoken normative commitments underlying scientific thought.

## Ernst Haeckel (1834–1919): art as an antidote to superstition

Few figures exemplify the convergence of science, philosophy, and art in the nineteenth century quite like Ernst Haeckel. A devoted advocate of Lamarck, Goethe, and Darwin, Haeckel was instrumental in popularizing Darwin’s theory of evolution in the German-speaking world. He identified and classified numerous species, introduced key biological terms (including “ontogeny,” “phylogeny,” and “ecology”), and is most recognized for his recapitulation theory, often referred to as the “biogenetic law.” This theory posits that the development of individual organisms reflects the evolutionary history of their species, encapsulated in the phrase “ontogeny recapitulates phylogeny.” Haeckel was also a gifted illustrator. His commitment to monism (Haeckel, 1892), the idea that all phenomena could be reduced to a single underlying principle, shaped both his biological theories and his artistic representations. For him, scientific illustration was not merely a tool for conveying knowledge but a means of acquiring knowledge (Breidbach, 2008; Forrester, 2020), revealing nature’s inherent symmetry, purpose, and beauty unified under a grand, hierarchical order.

For Haeckel, Darwin’s theory of descent was an epitome of common sense—a model of scientific clarity untainted by superstition or dogma. He saw it as the ultimate antidote to mythological thinking, thoroughly dispelling the remnants of pre-scientific thought. “Superstition and unreason are the worst enemies of the human race, while science and reason are its greatest friends,” he wrote in 1905 (Haeckel, 1905, p. 56). For him, the greatest challenge was to eradicate misconceptions and irrational beliefs that obscured the healthy reasoning with which humans were inherently endowed. He was particularly concerned with teleological explanations and arguments for intelligent design and divinely ordained purposes to explain organic life—the functionality of organs, their coordination within organisms, the adaptation of species to their environments, and the directionality of development. ‘We must grant,’ he conceded, ‘that at first glance the teleological theory seems to give the simplest and most satisfactory explanation of these purposive structures’ (Haeckel, 1912, Chapter XXV). However, for him, this was only because common sense was clouded by dogma, not because it was inherently flawed. Once liberated from such distortions and properly guided, common sense would become the very instrument that dismantled falsehoods.

Haeckel paid special attention to the doctrine of the soul’s immortality. For him, this belief was inseparable from teleological notions of purpose and design, as the soul was traditionally conceived as the nonmaterial force that directed bodies to fulfill their purposes. In *The Riddle of the Universe* (1901), written for the general public, Haeckel systematically addressed the standard ‘proofs’ of immortality, dismissing them as products of “poetic mysticism.” (Haeckel, 1901, p. 203). One by one, he addressed the theological, cosmological, moral, ethnological, and ontological proofs for the soul’s immortality, replacing them with physiological, histological, experimental, pathological, ontogenetic, and phylogenetic evidence ‘that prove the old dogma to be absolutely untenable.’ Confident in the march of scientific progress, he predicted that the issue of immortality would soon belong within the domain of ‘transcendental faith,’ no longer warranting serious scientific consideration (Haeckel, 1901, pp. 203–205).

Yet Haeckel’s interest in these beliefs extended beyond simply refuting them. He sought to understand why people, from the uneducated masses to the most cultivated intellectuals, clung so fervently to such ‘superstitions,’ viewing them as their ‘dearest possession and their most priceless treasure’ (Haeckel, 1901, p. 205). He saw them as “emotional cravings,” reinforced by the promise of eternal life and the grandeur of creationists myths (Haeckel, 1901, pp. 236–238). He understood that by challenging these convictions, he was not only confronting popular sentiment but also centuries of entrenched religious and philosophical authority. Nevertheless, he remained convinced that his reasoning was accessible to all—so long as they were willing to cast aside superstition and embrace “the clear sunlight of scientific knowledge” (Haeckel, 1880, p. 11)**.**

Haeckel himself, of course, was not immune to superstition. Like many of his contemporaries, he echoed the broader 19th-century anxieties about democracy and the political empowerment of marginalized groups. He actively contributed to the perpetuation of dangerous myths of racial superiority, asserting that certain races were inherently more advanced than others and that the psychic difference between the highest and lowest races was ‘much greater than is commonly supposed’ (Haeckel, 1901, p. 103; see Dombrowski, 2003; Gasman, 1971). By framing hierarchical racial distinctions as scientific fact, his work provided justification for eugenics and social Darwinism, leaving a troubling legacy of his interpretation of Darwinian theory.

Haeckel was convinced that his scientific worldview was grounded in pure, unclouded, healthy reasoning, but rather than dispelling dogma, he unwittingly reinforced it, believing he was laying bare the natural order with absolute clarity. He failed to see that the very biases he sought to overcome had shaped his own assumptions—assumptions he mistook for objective truth. To him, they confirmed the legitimacy of common sense, strengthening his belief that intuitive reasoning and scientific thought were naturally aligned.

From Haeckel’s point of view, his anthropogenic illustrations (Haeckel, 1874/1891) in which he depicted hierarchical order of human races were vital instruments for advancing what he believed to be true. Influenced by Humboldt, he held that aesthetic sensibility was essential to human knowledge as much as mechanistic explanation (Richards, 2008), shaping perception and belief through non-discursive modes of engagement. This demonstrates the power—and danger—of scientific imagery, which is always imbued with normative biases. The danger lies in mistaking aesthetic appeal for truth, assuming that what is beautifully ordered or commonsensically appealing must also be naturally given. It was precisely this recognition that led many 20th-century scientists to reject common sense as a reliable foundation for scientific reasoning.

For Haeckel, however, the belief that beauty offered a direct path to understanding, allowing a glimpse into ‘what holds the world together at its core’ (like Goethe’s Faust and his quest to find “*Was die Welt im Innersten zusammenhält*”) also shaped his infamous depictions of animal and human embryos. In his effort to support the biogenetic law, Haeckel produced drawings that exaggerated similarities between embryonic stages across species, often “enhancing” appearances, creating perfectly geometric structures, and adding visual embellishments to suit his theoretical claims. No major figure took such consistent liberties in imposing theoretical beliefs upon nature’s observable reality (e.g., Gould, 2000; for a different view, see Richards, 2009). However, his choice to prioritize idealized forms over strict biological accuracy reflected his broader belief in the didactic power of aesthetic coherence. For him, artistic embellishment was not a betrayal of nature but a means of revealing its deeper truths (Breidbach, 2008; Forrester, 2020).

While Haeckel in the nineteenth century believed that common sense, once liberated from the constraints of dogma and superstition, could reveal the true nature of the world, Waddington in the twentieth century viewed common sense as inherently flawed. He sought to transcend it and discover new ways to conceptualize the world through science and art.

## Conrad Waddington (1905–1975): art as a substitute for common sense

C. H. Waddington was an embryologist, geneticist, and evolutionary biologist whose work laid the foundations for systems biology, epigenetics, and evolutionary developmental biology. He developed a process and system-oriented approach, focusing on the cybernetic, multi-level feedback interactions that render developmental systems goal-directed (Peterson, 2016). Unlike reductionist explanations that isolate discrete mechanisms, Waddington emphasized the historicity and contingency of biological organization. He integrated multiple temporal and organizational scales—developmental, ecological, and evolutionary—viewing biological processes not as a collection of isolated parts but as dynamic systems embedded in historical and environmental contexts.

Contrary to the then-prevalent view, Waddington maintained that evolution and development cannot be decoupled. Previously, evolution was described in terms of distinct, independent hereditary factors (genes), which would randomly vary, leading to new traits in organisms. These traits were then either preserved or eliminated through natural selection. However, Waddington highlighted that we now understand these elements—hereditary factors, traits, and natural selection—as interconnected parts of an organized system. This system influences not only how genetic changes manifest as traits but also how natural selection operates within that framework (Waddington, 1969, p. 118).

Waddington’s approach was anything but commonsensical, and he made no effort to bring it closer to familiar ways of thinking. Rather than working within inherited conceptual structures, he sought to replace them entirely, introducing a new lexicon that would reshape how biological processes were understood. One of his key conceptual innovations was *canalization*, a term borrowed from Whitehead that described the capacity of developmental pathways to adjust in response to genetic and environmental variation, ensuring a uniform phenotypic outcome despite perturbations. This was not a mechanism in the traditional sense but a system-level reorganization that could occur across genetic, physiological, and behavioral levels. He described these canalized trajectories as *chreod*s (from the Greek for “necessary path”) and, for him, they were the most precise way to represent goal-directed biological processes as intrinsic patterns of teleological organization (Waddington, 1975, p. 223). By giving new meaning to old words and introducing unfamiliar ones, Waddington filled the world with new conceptual entities, disrupting the categories that common sense relied upon.

This radical reconceptualization was deeply shaped by his engagement with Whitehead’s process philosophy. For Whitehead (e.g., Whitehead, 1929), the fundamental units of nature were not atoms, forces, or energy but ‘*occasions of experience*’ or “*events*” (Whitehead’s term for what other people call ‘fact’ or ‘object’). Unlike common sense notions of objects as stable entities, an event—such as a stone or a cat—was a time-extended occurrence that contained within it references to every other event in the universe. Each event was a knot in an infinitely complex network of spatial and temporal relations, constantly reshaping itself as it unfolded. Whitehead further destabilized familiar categories by describing events as having *feelings*. He used this notion to capture how an event absorbs aspects of other events into itself in a process he called *prehension*. By adopting and adapting Whitehead’s conceptual framework,^3^ Waddington likewise displaced mechanistic descriptions with a vocabulary that emphasized fluidity, interdependence, and historical contingency over fixed structures and causal chains.

Indeed, it seems that for both Whitehead and Waddington, their ideas gained strength precisely through their distance from common reason. Having lived through two world wars in an era of profound disillusionment with inherited intellectual traditions, Waddington likely saw appeals to common understanding as perilous. His adoption of Whitehead’s philosophy and his own development of a technical lexicon were not just ways of describing biological processes—they were acts of epistemic rebellion. In replacing common sense with a new world of concepts, he signaled a deliberate (if not entirely conscious) refusal to recognize common understanding as an authority to which science owed any allegiance.

“The world of billiard ball atoms existing at definite times in simple three-dimensional space dissolved into the esoteric notions of quantum mechanics and relativity,” writes Waddington (1969, p. 2). These changes “made it impossible to accept the conventional view of the world as an assemblage of material ‘things,’ which exist in space but endure through time” (Waddington, 1969, p. 113). With the realization that fundamental particles do not follow each other in strictly determined causal sequences, “commonsense has taken a terrific beating” (Waddington, 1969, p.109). For both science and art, multiple spatiotemporal frames now existed, with “no way of determining, indeed no meaning in asking, which of these frames is ‘correct’” (Waddington, 1969, p. 13). Depicting nature, as seen from our eyes, was no longer an accurate representation of it at all, leading Waddington—in stark opposition to Haeckel—to consciously ‘retreat from likeness’ (Waddington, 1969, p. 1).

Waddington sought substitutes for common sense and outdated modes of reasoning. He believed that emerging technologies, particularly computational methods, could provide more reliable tools for scientific inquiry. Interestingly, he also considered art as an alternative to common sense. In *Behind Appearances: A Study of the Relations Between Painting and the Natural Sciences in This Century* (1969), he explored the mutual influence of art and science in the twentieth century, emphasizing how both domains enriched one another. Like our aim in this paper, Waddington sought to uncover the *Zeitgeist*—the cultural climate—by tracing the resonance between visual art and scientific thought. Some artists, he believed, absorbed scientific ideas “through the pores” and infused them into their work, not by explicit illustration but through a deeper incorporation of the scientific mindset (Waddington, 1969, p. 239). Scientists would then turn to these artists for a deepening of consciousness, finding in painters a reflection of the scientific spirit. This exchange, he suggested, allowed certain less easily articulated aspects of scientific inquiry to be ‘shown forth’ through artistic expression (Waddington, 1969, p. 155). This approach created an aesthetic engagement with scientific ideas that was far removed from common sense, instead inviting viewers into a world governed by abstract, often counterintuitive principles.

For Waddington, the interplay between time, space, mass, and the observer’s role in both scientific and artistic creation was central to the *Zeitgeist* of the twentieth century. His notion of *Zeitgeist* reflected the pluralism inherent in a systems-based view of nature, where different organizational levels constantly interact. He believed that a systems-oriented worldview would become a dominant theme in the future of both science and art (Waddington, 1969) and summarized his understanding of 20th-century thought in four key insights:

“(a) The epistemological foundation. The observer does not wholly make what he observes, but his intrinsic character colours it. There is no strict objective-subjective dichotomy. The painter is *in* his painting, the scientist is *in* his science.

(b) Chance plays a role amongst the fundamental mechanisms.

(c) Everything ‘has a feeling for’ (prehends) everything else; things have fuzzy edges.

(d) On a more down-to-earth level: we live in surroundings and conditions that we ourselves make,^4^ not in any state of nature that we have to accept in its entirety.” (Waddington, 1969, p. 240, his emphases).

Waddington’s scientific image—the world “behind appearances” (the title of his 1969 book)—was fundamentally different from everyday observation. This hidden world required imagination, and he saw art as instrumental in revealing it. No longer was nature itself the primary subject of representation; instead, scientific explanations became objects of aesthetic appraisal. For Waddington, living in an environment shaped by human intervention was central to the scientific mindset, and because “the scientist is in his science,” this world had to be actively constructed. This, however, was precisely Arendt’s concern: that in modernity, the act of knowing had become inseparable from making and that scientific thought was increasingly governed by the kind of fabrication associated with manufacturing. She feared that modern science’s emphasis on process over product had diverted attention from the metaphorical “table” of a shared world, undermining its function as a stable platform for political action.

Waddington also used art to stimulate his scientific imagination and, as he put it, to “loosen the joints of his psyche” (Waddington, 1969, p. 242). In his *Organisers and Genes* (1940), he included as a frontispiece a painting by John Piper to illustrate how genes and environmental factors interact to guide development along branching pathways. The painting shows an abstract, layered landscape of curved ridges and deep, shadowed valleys carved into the terrain. The overall impression is both architectural and organic: an undulating surface that hints at branching possibilities without explicitly mapping them. Later, in his *The Strategy of the Genes* (1957), he added a schematic depiction where valleys represent stable developmental states, while ridges mark barriers or thresholds that must be crossed to transition into alternative trajectories.^5^ Reflecting on the role of artistic representation in science, Waddington later wrote:

“… many people including myself, find that it is often useful and enlightening to have visual illustrations of ideas. This book is therefore provided with a large number of diagrams. These are not intended to express facts about quantities or things […] They are strictly illustrations of ideas, and their purpose is to stimulate your imagination to seize the gist of what the idea is about” (Waddington, 1977, pp xii–xiii).

He thought visual metaphors and abstract illustrations were better suited for making sense of an increasingly dynamic scientific reality that embraced complexity, process, and interaction over fixed categories. This, however, came at a price—the price, as Arendt warned, of alienation: both between individuals and the world they inhabit, and between the scientific community and public trust.

## Ginsburg and Jablonka: art as a mode of negotiation

In the twenty-first century, science finds itself in a precarious position. It must uphold rigorous standards and build on a body of knowledge that has driven decades of progress, yet it faces mounting pressure to reclaim its role in shaping a shared world and maintaining public trust. This challenge has become especially acute in an era of fragmented identities, clashing narratives, and competing worldviews, where science itself is increasingly met with skepticism. Scientific authority also faces internal questioning, as many scientists acknowledge the historical and cultural contingencies of their normative frameworks and recognize that their jurisdiction is not self-evident but historically situated. The crisis, then, is not only about science’s standing in the public sphere but also about its own internal reckoning with its epistemic foundations.

This very reckoning is part of how Ginsburg and Jablonka use art in their work. They knowingly use it as a way to project—both outward onto their readers and inward onto themselves—the very assumptions on which their scientific reasoning rests. Between the demands of rigor and the need for shared meaning, they offer art as an avenue for negotiation, where the less explicit aspects of their work can be grasped through non-conceptual modes of communication.

Ginsburg and Jablonka explore how consciousness and goal-directed behavior evolved, arguing that the emergence of unlimited associative learning (UAL) marked a major evolutionary transition (Ginsburg & Jablonka, 2019). Unlike basic forms of learning, UAL involves the flexible formation of an open-ended range of associations between different types of stimuli, actions, and outcomes. It requires novelty, generalization, and the ability to revise or reverse learning in new contexts. Such flexibility, they argue, depends on the integration of perception, memory, and motivation into a unified experiential field, making UAL a plausible indicator of a minimal form of subjective experience. It allows such animals to assign meaning to their experiences based on felt needs, past learning, and future anticipations.

With the evolution of consciousness, goal-directed behavior gained a new depth (Jablonka & Ginsburg, 2023). Organisms with UAL integrate perceptual and affective representations into their decision-making, continuously updating stored sensory and motor memories to shape neural pathways and guide intentional behavior. Ginsburg and Jablonka extend the processual perspective to evolutionary dynamics, drawing on Waddington’s perspective while expanding the scope of selection and heredity.

This perspective may run counter to common sense. However, for Ginsburg and Jablonka, the phenomenon they seek to explain—the evolution of intentional, motivated action—cannot, and should not, be severed from our intuitive sense of agency. Consciousness, in their view, is not merely a byproduct, or epiphenomenon, of biological processes. Instead, they see it as emerging from the ongoing interplay between biological mechanisms and their phenomenological interpretation—each shaping the other.

This approach directly engages with Arendt’s concerns about modern science’s turn toward abstract accounts of reality and the risk of severing its connection to the “common world”—the shared, intersubjective space where meaning is negotiated. When scientific claims are removed from lived experience, they become estranged from the very world they seek to explain. Ginsburg and Jablonka’s conception of consciousness resists this drift. By insisting that consciousness cannot be understood without attending to its subjective, experiential dimension, their approach keeps scientific inquiry tethered to the realm of lived meaning.

Ginsburg and Jablonka’s implicit commitment to such a response is also evident in their public outreach, particularly in *Picturing the Mind: Consciousness through the Lens of Evolution* (2022). Zeligowski’s paintings in this book are not meant to illustrate scientific ideas but to actively interpret them and reflect on the very act of seeing and understanding. It is as much her journey into the scientific world as it is a gesture toward others. Unlike Waddington’s abstract forms, she maintains a steadfast connection to nature, preserving the integrity of recognizable, real-world forms. Yet she refrains from dictating how these forms should be read: the compositional structure and unfolding narratives of many of her paintings remain deliberately open (As Fig. 1 clearly shows). This openness is less a matter of ambiguity than of orientation—an active positioning of oneself in a way that echoes Arendt’s idea of “sharing-the-world-with-others”. In its own unique way, her art challenges science’s monopoly on truth-telling. It seeks to decontextualize and defamiliarize—to create new atmospheres and stimulate thought.

**Fig. 2:**
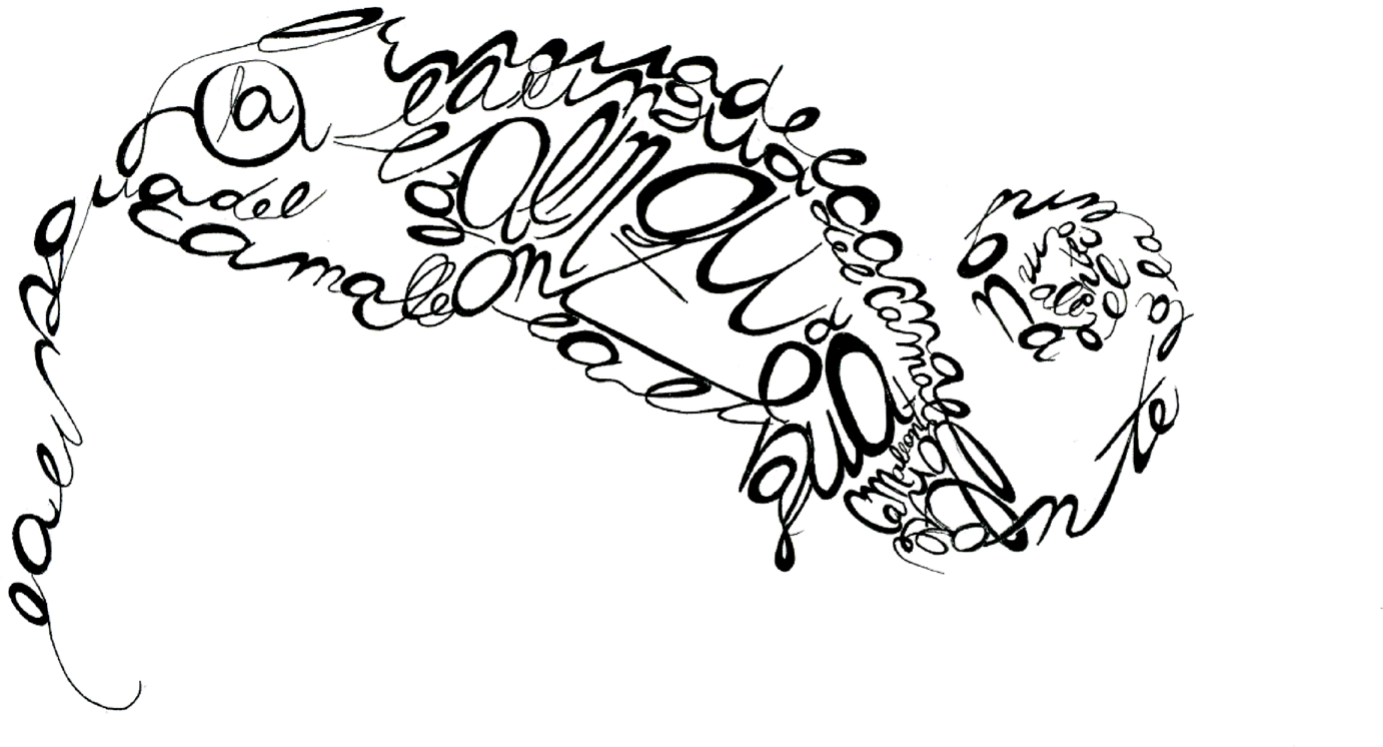
*la lingua del camaleonte* Permission given by the artist (@ Anna Zeligowski)

This approach also places Zeligowski in a position to engage with inherent limitations in Ginsburg and Jablonka’s project, one of which concerns the role and limits of language in understanding minimal consciousness. Drawing on Aristotle’s notion of the “sensitive soul,” Ginsburg and Jablonka locate the ‘second teleological transition’ in the emergence of subjectivity: the ability to experience without the reflective self-awareness of the rational soul. For them, consciousness need not be conscious of itself and there can be consciousness without explicit awareness of the mind-world chasm and without second-order linguistic representations. Such a pre-linguistic consciousness cannot be separated from the act of experiencing, and to be faithful to the phenomenon, Zeligowski must avoid splitting herself into an acting self and an observing self. Failing to do so risks losing sight of the phenomenon itself and disregarding the very question of what consciousness truly is. Zeligowski must, therefore, explore the limitations of (Ginsburg and Jablonka’s) language and reimagine its role. Still, she knows she cannot set aside the discursive structures of her human mind, as they are shaped by language itself and inseparable from her way of thinking and perceiving. The use of calligrams —text arranged visually to create images that evoke their meaning— is an interesting strategy she adopts in some of her paintings. It transforms language from a tool of abstraction and rigid categorization into a fluid form that blurs the boundaries between concept and representation, shifting it from a barrier to a participatory medium. Zeligowski’s choice to reintegrate language speaks to the inherent difficulty of humans endowed with language to understand the soul of those who do not have language but are conscious, sensitive, subjectively experiencing beings (Fig. 2).

**Fig. 3:**
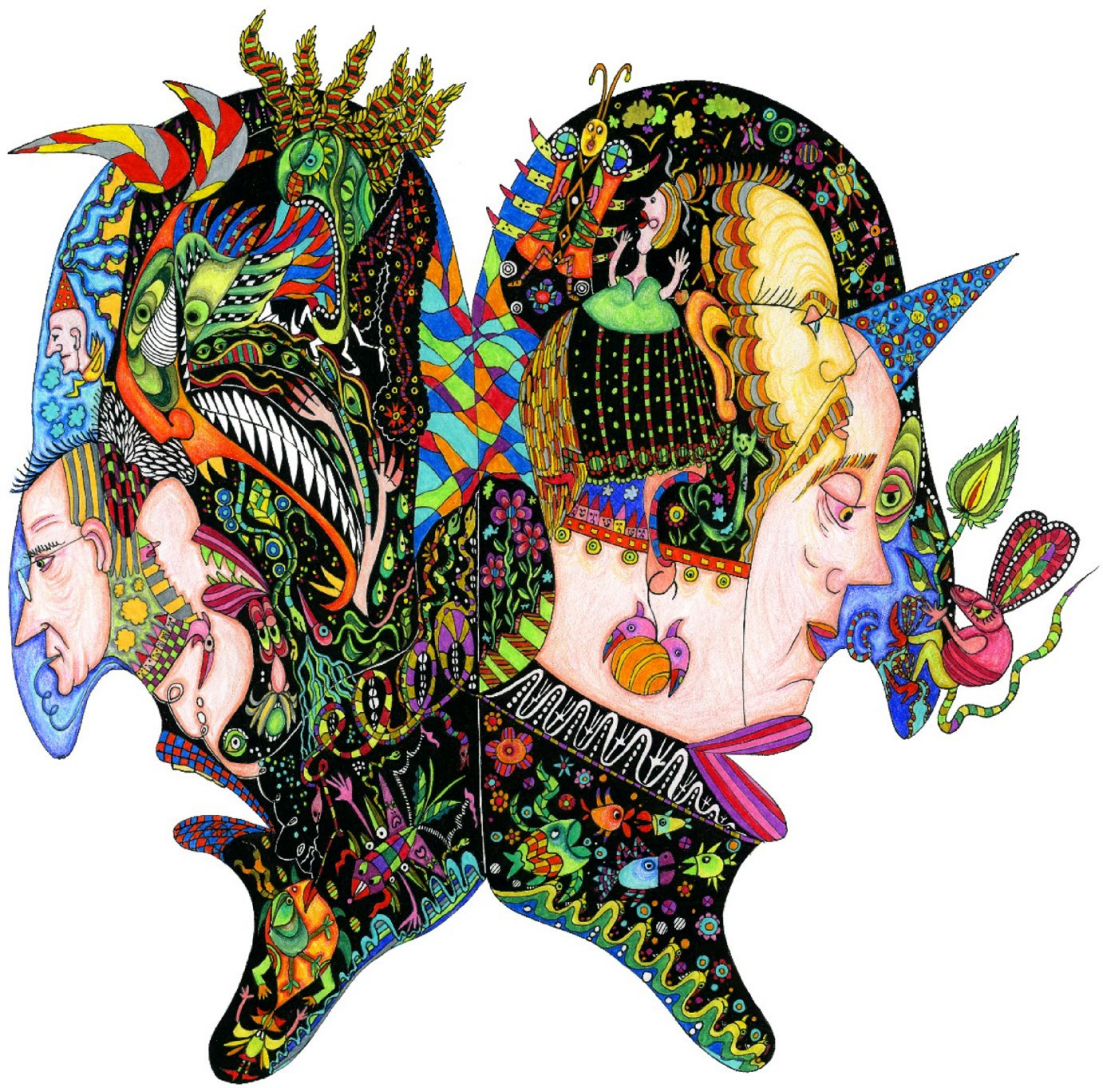
*‘A torn soul’* Permission given by the artist (@ Anna Zeligowski)

What also sets Zeligowski’s work apart from the artistic strategies of Haeckel and Waddington is its engagement with social and ethical concerns, extending beyond the scientific domain to forge a connection with the everyday world shared by scientists and laypeople alike. For instance, in Zeligowski’s depiction of ‘a torn soul,’ where Ginsburg and Jablonka criticize Freud, Lorenz, and Koestler for reducing the human psyche to simple dichotomies (good and evil, yin and yang, Eros and Thanatos), Zeligowski’s painting is of something that looks like a butterfly. On each of its wings, human heads face opposite directions, but their expressions cannot be easily categorized as rational or emotional opposites. This ‘butterfly’ is now deformed. The image illustrates how such binary views not only fall short of a proper scientific understanding but also distort nature itself (leaving the butterfly unable to exercise its natural right to fly) (Fig. 3).

Here, as in all of Zeligowski’s paintings, she highlights that scientific understanding cannot rely on oversimplified assumptions, yet it also need not lose itself in obscure scientific language. There is a third way of knowing and engaging with the world, one that embraces both clarity and depth, allowing knowledge to be explored through artistic insight. In doing so, she creates a space where the complexity of nature can be grasped not just intellectually but also intuitively, offering a more immersive and holistic experience of scientific inquiry.

## Judgment, sensus communis, and the universal voice

The examples we have explored demonstrate that while scientists’ attitudes toward human rationality and everyday reasoning have shifted dramatically over the last two centuries, they have consistently placed faith in an underlying aesthetic sensibility. This sensibility has not always appealed to common sense in the popular register—as a rational, judicious faculty of discernment—but it has, time and again, drawn upon what Kant called *Gemeinsinn*: the aesthetic common sense which, in his view, is more deserving of the name *sensus communis*. Such an aesthetic awareness, for Kant, is the human capacity to discern coherence in what would otherwise remain beyond conceptual reach.

We turn to Kant not because scientists consciously or unconsciously appeal to his aesthetics when depicting their work, nor because their aesthetic choices can be fully explained within his philosophical system. What makes the Kantian approach relevant here is his analysis of aesthetic judgment—particularly his concept of *sensus communis*—which offers a way for understanding how aesthetic representations can both reflect and shape normative commitments.

Kant’s insists that aesthetic judgments, though subjective and conceptually indeterminate, lay claim to *universal validity* (5:239^6^). This allows us to understand how a particular image can stand for, or gesture toward, something universal and how it can lay claim to normative significance. Our argument that aesthetic representations in science provide us with access to the normative background of scientific inquiry depends, at its core, on this Kantian idea. It is this peculiar quality of aesthetic judgments —subjective yet communicable, conceptually indeterminate yet normatively potent—that makes such representations capable of revealing the implicit commitments that structure collective thought.

Moreover, Kant’s model allows us to see how such images can exert normative force and how they can do more than reflect historically contingent norms. They can call them into question, reshape what is taken to be intelligible, and open space for normative reorientation. In moments of genuine aesthetic experience, viewers confront possibilities that transcend their current normative conditions: if a particular can stand for the universal, it can also *reimagine* it. It can destabilize existing claims to universality and open new, universally valid horizons. Art here does not merely represent the world—it interrupts it, reframes it, and its power lies in shifting the very ground of judgment, exposing the contingency of dominant narratives, and altering the conditions of sensibility itself.

In each of the cases we explored, scientists have harnessed the normatively authoritative power of aesthetic references but used it differently; Haeckel used it against superstition, Waddington to create a world ‘behind appearance,’ and Ginsburg and Jablonka to negotiate the terms of public trust. In each case, aesthetic representations became sites where normative predispositions are not only reflected but potentially transformed.

While the ‘common human understanding,’ for Kant, is tied to discursive reasoning and conceptually determined judgment, *Gemeinsinn*, the aesthetic common sense, refers to the human capacity to find coherence in the absence of determinate concepts (5:231). This coherence is, therefore, *felt* rather than *cognized* (5:219). It emerges when imagination and understanding enter what he calls “free play” (5:217). In contrast to ordinary cognition, where understanding guides the imagination in subsuming particulars under universal concepts (5:179), in aesthetic judgment, understanding does not dictate which concept an intuition must fall under. Instead, it provides a kind of formal openness, allowing the imagination to explore and generate a felt sense of unity—purposiveness without purpose (5:226). What makes this form of judgment distinctive, for Kant, is that it presupposes a *sensus communis*—a subjective principle that allows us to judge by feeling rather than by concepts, though nonetheless with universal validity (5:238). The feeling of pleasure we experience in such judgments is not the satisfaction of recognition under concepts but an explorative, reflective pleasure that arises from finding non-conceptual unity.

This is precisely the kind of conceptual indeterminacy we seek when discussing normative, implicit, or tacit orientations that guide thought and practice without being explicitly articulated. Scientific reasoning, even at its most rigorous, is always embedded in a background of assumptions, values, and expectations. Every scientific body of knowledge says more than what is explicitly stated—it is a discipline in the normative sense of the word. It implicitly dictates what is worthy of being called knowledge and what constitutes scientific understanding. These implicit dimensions—what a discipline renders intelligible, what it excludes, and what it takes for granted—cannot be fully grasped through determinate concepts. Instead, we find ourselves already situated within a normative space of felt responses and shared practices. Aesthetic judgments operate on the same level of conceptual indeterminacy. They speak the same ‘non-language’ and reveal coherence where no rules can be clearly stated. In doing so, they help us register the implicit contours of our normative disposition.

Following Inbar (2014), we suggest that aesthetic judgments make normative demands of us, and aesthetic awareness is an attunement to those demands—to the felt alignment between imagination and understanding. When we experience the non-conceptual unity characteristic of aesthetic judgment, we become aware of the normative background that makes such unity intelligible. Importantly, we become attuned to the fit—or mismatch—between our normative dispositions and those that the object demands of us. Aesthetic experience, in this way, illuminates not only how we see but how we are disposed to see and how those dispositions might be reconfigured. Aesthetic awareness can thus introduce a rupture—a productive dissonance—allowing us to push back against inherited norms. This is the critical and transformative potential of aesthetic experience (and the revolutionary power of art): It does not merely reinforce our normative world—it renders visible the unspoken norms that shape our judgments while opening a space in which they can be questioned, disrupted, or transformed.

However, such normative transformation would not be possible unless we take our judgment to be universally valid and unless we take into account (a priori) how others might judge (5:293) and “think from the standpoint of everyone else” (5:294). Judgment can have such power only if it speaks with a “universal voice” (5:216). This is also why Arendt thought that any meaningful response to the crisis of alienation must begin with the revival and cultivation of the Kantian *sensus communis*. She regarded it as ‘the political sense par excellence’ (Arendt, 1994, p. 318), enabling individuals to orient themselves in the public realm (Arendt, 1961, p. 221). Judging and its subjective universality, she argued, is “the most important activity in which this sharing-the-world-with-others comes to pass” (Arendt, 1961, p. 221) because it safeguards “the people” without collapsing the differences between them.

## Discussion

We have argued that aesthetic representations in science provide access to the normative background within which they are embedded. They highlight overlooked, underemphasized, and often only vaguely apprehended features and patterns that shape scientific reasoning. Operating within a non-conceptual realm, they give form to what otherwise remains beyond conceptual reach and stances we typically take for granted.

One such stance concerns the extent to which science can and should appeal to common human understanding. We have focused on this assumption not only because it has profoundly shaped the trajectory of science over the past two centuries but also because it reflects how scientists perceive their public role. The ways in which scientific reasoning has relied on, distanced itself from, or negotiated with ‘the common understanding’ reveal deeper shifts in epistemic commitments—shifts that aesthetic representations make accessible.

In this view, the role of aesthetic judgments in science is to shift the center of epistemic gravity to non-conceptual modes of reasoning. They reveal how conceptual and non-conceptual forms of knowledge not only coexist but actively support and enrich one another. In doing so, as Elgin (2002) notes, “they sensitize us to, and sometimes call into question the validity of, stances we typically take for granted” (p. 8).^7^ In this sense, aesthetic judgments are not merely about the object of appreciation but about the judging subject (Longuenesse, 1998, 2006). They confront us with our existing normative commitments, making them visible and available for critique.

Our approach diverges from recent scholarly work that has considered the role of aesthetic judgments in science. Broadly speaking, these approaches take two opposing views. Some suggest a pre-cognitive role for aesthetic judgments, where aesthetic experiences are, in some sense, a condition for acquiring scientific concepts. Others have instead taken a meta-cognitive approach, suggesting that aesthetic judgments are dependent on theoretical beliefs.^8^

Breitenbach (2021) represents the first of these perspectives, proposing that while aesthetic judgments are insufficient for cognition,^9^ they play a central role in the search for determinate cognition and in the creative process of discovery, of formulating new concepts, and generating new theories. On her account, aesthetic experiences present unities that the subject judges as suitable for human understanding, even if the understanding does not yet categorize this unity under any particular concept. Aesthetic judgments then direct attention to potential objects of cognition by signaling what might be valuable when subjected to processes of justification.

While we agree that aesthetic judgments play a role in scientific ingenuity, we argue that true innovation requires an evaluation of our existing normative commitments to which aesthetic judgments provide access. Because normative frameworks function as self-contained, incommensurable systems, meaning remains enclosed within them, inaccessible through language-based analysis.^10^ On our account, aesthetic awareness offers a foot outside our existing normative dispositions and an alternative, normatively valid standing from which our normative commitments can be critically examined. It is not just a question of highlighting potential objects of cognition but of exposing the epistemic and normative structures that condition our judgments. Aesthetic judgments thus enable a deeper kind of epistemic reflexivity, an ability to step outside the assumptions embedded in our current conceptual schemes, making possible a form of innovation that is not just generative but transformative.

As for meta-cognitive approaches, some take aesthetic judgments to be second-order, self-reflective responses to cognitive judgments (e.g., Breitenbach, 2013, 2020). On this view, aesthetic judgments are an awareness of the very fact that we understand—an awareness of the suitability of our intellectual capacities for understanding the world. Breitenbach (2013) grounds this claim in Kant’s assertion that nature is “subjectively purposive,” arguing that the essence of aesthetic experience lies in recognizing that nature lends itself to being judged by us (5:193). Aesthetic judgments, then, concern the judging subject rather than the object, and they depend on cognitive judgments because one must first grasp what one’s cognitive faculties can achieve in order to appreciate this achievement aesthetically. Not every scientific theory is beautiful on this account since “even if we gain understanding in a particular case, it is a further question whether or not we become reflectively aware of this harmony” (Breitenbach, 2013, p. 95).

We agree that aesthetic judgments are second-order, self-reflective judgments about ourselves as judging subjects. We also agree that it is only because we gain an understanding of the world that we also gain an understanding of ourselves as knowing subjects, and that we can understand without being aware of the fact that we also indeterminately pick up on something that goes beyond concepts. However, we argue that aesthetic judgments offer more than a mere awareness of the fact that we can understand. They have content of their own. It is not simply the fact that something is subjectively purposive that gives rise to aesthetic pleasure, but its purposiveness itself—the undetermined (rather than determined) regularity it manifests. If the “further” appreciation Breitenbach describes amounts only to recognizing that something has been purposive for our cognitive faculties, then it is unclear why such appreciations should not be classified as intellectual pleasures rather than aesthetic ones. Intellectual pleasure does not stem from subsuming particulars under concepts but from recognizing that we have the capacity to do so.

On our account, aesthetic appreciation does not merely affirm our ability to understand; it reveals what renders our understanding meaningful by shifting epistemic weight to the normative background that our judgments presuppose. Not every scientific theory is beautiful, not simply because we are sometimes distracted from recognizing the cognitive accomplishment we have made, but because we can understand something without being aware of what it normatively commits us to. One can understand, for example, Haeckel’s or Waddington’s theories and easily overlook their implicit implications regarding the relationship between science and common sense. Aesthetic engagement, however, makes such commitments more difficult to ignore.

Other meta-cognitive approaches (e.g., Carlson, 2002) argue that a deep and appropriate aesthetic appreciation of nature requires scientific knowledge. In this view, aesthetic judgments depend on theoretical beliefs to direct attention, ensuring that perception is properly informed. An interesting example of this approach is Parsons (2006), who worried that Carlson’s’highly theoretical beliefs’ were “incompatible with aesthetic appreciation, which is, by all accounts, appreciation of the sensuous” (p. 168). He, therefore, pursued an account that could ‘connect’ theoretical knowledge to sensuous experience. Building on the theory-ladenness of observation, Parsons argues that scientists, through training and habituation, develop the capacity to *see* nature through their background knowledge—perceiving theoretical facts directly rather than merely inferring them (Parsons, 2006).

However, if aesthetic appreciation is wholly governed by cognition, then the experience risks becoming an extension of theoretical knowledge rather than an engagement with the object itself. This model dictates the content of appreciation by infusing perception not only with theoretical beliefs but with the normative commitments that underpin those beliefs. It risks neutralizing the power of aesthetic experience and the possibility of seeing differently. If observation is always already structured by background beliefs, then aesthetic experience ceases to be a space for transformation.

One of the central roles of aesthetic engagement—especially in relation to scientific representations—is precisely its capacity to bring to the surface the norms that shape scientific reasoning and, crucially, to create the possibility of seeing things differently. If aesthetic experience is to retain its transformative power, it must not be wholly absorbed into conceptual frameworks that structure observation. Instead, it must remain a space where implicit commitments can be questioned, and potentially reoriented. Transformation requires distance, a kind of estrangement from theoretical beliefs that aesthetic representations of those very beliefs can provide.

Moreover, the assumption that greater knowledge necessarily enhances aesthetic appreciation is problematic. Scientific understanding can sometimes impede rather than enrich aesthetic engagement. When epistemic commitments exert too strong a pull, they can dull one’s ability to respond to what the object normatively demands and impede aesthetic engagement. Aesthetic appreciation, in our view, requires not just seeing *through* knowledge but seeing *beyond* it by accessing the normative structures that govern perception. It is this access, we believe, that fosters aesthetic pleasure, and it is what distinguishes aesthetic appreciation as a unique kind of epistemic act.

Currie (2023) offers a perspective that aligns more closely with our approach, particularly in connecting aesthetics and knowledge in science through the concept of ‘epistemic engagement.’ While we do not see aesthetic judgments as knowledge-directed processes in the strong or cognitive sense, we agree that aesthetic judgments are ‘co-opted towards epistemic ends.’ When Currie describes how various social practices and shared experiences ‘attune’ scientists to a paradigm and considers skills, perspectives, open questions, expectations, and motivations, he points to the implicit knowledge that drives scientific practices. This attunement demonstrates how aesthetic sensibility influences every aspect of scientific practice and why periods of scientific revolution can be seen as moments of ‘aesthetic raptures’ (McAllister, 1999). These raptures, we have argued, are between our old and new normative commitments, and true scientific ingenuity necessarily involves being “attuned” to the weight of our normative commitments.

By engaging with aesthetic forms, scientists navigate and negotiate the boundaries of conceptual understanding itself, reflecting and reshaping the very conditions of intelligibility. To aesthetically appreciate what an object normatively demands, one must be aware of the conceptual terrain in which it operates. In this sense, aesthetic judgments are meta-cognitive. Yet, in the course of scientific progress, the aesthetic shift in epistemic gravity can unsettle normative commitments, allowing the free play of the faculties to generate new normatively valid ways of thinking that can later serve as a foundation for concept formation.

## Footnotes

1 We will use the terms ‘common sense,’ ‘common understanding,’ and ‘the good sense of the people’ interchangeably, distinguishing them from Kant’s *sensus communis* only in Sect. 6.

2 McAllister (2023) observes that in 1917, Whitehead still held that science’s ultimate task was to satisfy common sense thought, asserting that common sense “is the datum from which it starts, and to which it must recur” (Whitehead, 1917, p. 62).

3 For a discussion of Whiteheads influence on him, see Waddington (1969, 1975).

4 Waddington refers here to both nature and science. He discussed different aspects of what we call today ‘niche construction’ (Waddington, 1959).

5 Peterson (2016) Squier (2017) Nicoglou (2018) and others explored the epigenetic landscape metaphor and its influence on biology and other disciplines.

6 References to Kant’s Critique of Judgment (1790) are to volume and page of the *Akademie edition: Kants gesammelte Schriften*.

7 Elgin (along with Goodman) holds a different conception of cognition and understanding than Kant, incorporating interpretive practices. We draw on Elgin’s perspective specifically to highlight how aesthetic experiences can unsettle normative commitments because we agree with her broader view that subsuming particulars under concepts is just one way of seeking understanding, though not in a Kantian sense. Like Elgin (1996), we believe that we ascribe meaning to symbols, metaphors, and narratives, even though they do not 'augment our stock of literal truths' (p. 170).

8 For a comprehensive account of pre-cognitive, multi-cognitive and meta-cognitive interpretations of Kant’s notion of the free-play, see Guyer (2006).

9 From A Kantian view, these approaches suggest that the free-play fulfill the first two syntheses required for cognition without the final synthesis of subsuming intuitions under a determinate concept. This pre-cognitive approach is supported by several Kantian scholars (e.g., Ginsborg, 1997; Makkreel, 1990).

10 This has famously led Kuhn, Feyerabend, Rorty, Carnap, and others to maintain that the transfer of allegiance from one normative vocabulary to another during a paradigm shift is not a rational choice and is better described as a religious conversion, gestalt switch, or as simply a matter of pragmatic preference.
